# A Study of Adverse Drug Reactions to Antiepileptic Drugs in Pediatric Epileptic Patients From North India

**DOI:** 10.7759/cureus.96343

**Published:** 2025-11-07

**Authors:** Puneet Kaur, Anjleen Kaur, Manvir Kaur, Harjinder Singh, Jasbir Singh

**Affiliations:** 1 Pharmacology, Government Medical College and Rajindra Hospital, Patiala, IND; 2 Public Health, Johns Hopkins Bloomberg School of Public Health, Maryland, USA; 3 Pediatrics, Government Medical College and Rajindra Hospital, Patiala, IND

**Keywords:** adverse drug reactions (adr), antiepileptic drugs (aed), epilepsy in children, north india, side‐effect

## Abstract

Objectives: The primary objective of this study was to evaluate the various adverse drug reactions (ADRs) associated with antiepileptic drugs in children. The secondary objective was to analyze the distribution of these ADRs according to gender, age group, type of epilepsy, and treatment modality.

Materials and methods: An observational cross-sectional study was conducted in the pediatric outpatient department of a tertiary care hospital. A total of 100 eligible patients, who fulfilled the inclusion and exclusion criteria, were enrolled in the study after obtaining informed consent. The prescription details and ADRs were collected using a pre-designed proforma and the Pediatric Epilepsy Side-effect Questionnaire, respectively. The collected data were then analyzed using descriptive statistics and the chi-square test, where appropriate. The p-value <0.05 was considered statistically significant.

Results: A total of 67 ADRs were reported in 42 participants, predominantly in males (71.43%). Polytherapy (75%) was associated with a higher number of ADRs (p=0.002). Patients treated with valproate (monotherapy group) and valproate with clobazam (polytherapy group) reported a greater number of ADRs (57.14%, 72.73%).

Conclusions: The study highlights that ADRs are more common in patients on conventional drugs like valproate, either as monotherapy or polytherapy. Use of newer drugs by physicians and reporting of ADRs by patients should be encouraged for the comprehensive management of childhood epilepsy.

## Introduction

Epilepsy is a common neurological disorder characterized by recurrent, unprovoked seizures, affecting individuals across all age groups. In 2021, an estimated 51.7 million individuals globally were living with active epilepsy, with an age-standardized prevalence of 658 per 100,000 population. More than 80% of this global burden is concentrated in low- and middle-income countries [1]. In India, it affects 0.5% to 1% of adults and 0.35% to 0.55% of children, with childhood epilepsy having a prevalence rate of 6.24 per 1,000 population [2]. Antiepileptic drugs (AEDs) are the mainstay treatment of epilepsy, which are further classified into older/conventional and newer categories based on their market introduction, with the cutoff year being 1991. Conventional drugs (such as phenytoin, carbamazepine, valproic acid, and ethosuximide) are commonly used as first-line treatments and are generally more affordable. In contrast, newer AEDs (such as gabapentin, lamotrigine, vigabatrin, topiramate, tiagabine, and zonisamide) tend to be more expensive [3]. The AEDs have a narrow therapeutic index and are associated with multiple adverse drug reactions (ADRs), thus raising significant safety concerns [4]. Most of the data on AED-related ADRs that are currently available have come from studies conducted in adults [5]. Thus, of all the age groups, children are considered particularly susceptible to ADRs due to limited data from drug development processes and the frequent off-label and unlicensed use of medications [6]. Further, various social, cultural, and demographic factors can play a role in the occurrence of different ADR due to AEDs in children across different regions of the world. Notably, there has been a dearth of studies focusing on the occurrence of ADRs in children using AEDs, particularly in the North Indian population. To address these gaps, a cross-sectional study was conducted in 100 eligible pediatric patients visiting the outpatient department of a tertiary care hospital. The primary objective of the study was to evaluate the various ADRs associated with AEDs in children, while the secondary objective was to analyze the distribution of these ADRs according to gender, age group, type of epilepsy, and treatment modality.

## Materials and methods

This observational cross-sectional study was conducted over a period of one year in the pediatric outpatient department of Rajindra Hospital, Patiala, following approval from the Institute Ethics Committee of Government Medical College Patiala (approval number: BFUHS/2K21p-TH/14873). A total of 100 eligible children (sample size calculated using standard normal variate (Z) (at 1% type 1 error) as 2.576, expected proportion (p) based on previous studies as 0.624%, and absolute error or precision (d) as 2%) were enrolled after fulfilling the inclusion and exclusion criteria during the period of one year [2]. Written informed consent was obtained from parents, and assent was collected from children older than seven years. Children of either gender, aged <18 years, diagnosed with epilepsy (idiopathic or symptomatic), and who had received antiepileptic treatment for at least 3 months were included in this study, while the children with known intellectual disability, global developmental delay, autism, attention deficit hyperactivity disorder, cerebral palsy, or other pre-existing chronic illnesses such as renal or liver disease were excluded from the study.

Demographic data and complete prescription details of all participants were documented using a pre-designed prescription proforma. The ADRs were assessed during clinic visits using the Pediatric Epilepsy Side-effect Questionnaire (PESQ), which categorizes AED side effects into five domains: cognitive, motor, behavioral, general neurological, and weight [7]. The permission to reproduce the PESQ was obtained (license number: 5444210984904). Further, any other ADRs reported by parents were also recorded. Each patient was evaluated only once.

The collected data were entered into Excel (Microsoft Corp., Redmond, WA, USA) and analyzed using descriptive statistics in SPSS Statistics version 22.0 (IBM Corp. Released 2013. IBM SPSS Statistics for Windows, Version 22.0. Armonk, NY: IBM Corp.). The chi-square test was used to analyze categorical data, with a p-value <0.05 considered statistically significant.

## Results

In our study, we observed a male predominance (55%, n=55) compared to females (45%, n=45). The majority of patients (55%, n=55) belonged to the 6-11 years age group, and generalized epilepsy (78%, n=78) was the most common type of epilepsy observed. On average, 2.35 drugs were prescribed per patient. Other demographic and clinical details are represented in Table 1.

**Table 1:** Demographic and clinical characteristics of study participants SD: standard deviation

Sr. no.	Characteristic	Category	Number of patients (%age)
1.	Gender	Male	55 (55%)
		Female	45 (45%)
2.	Age group (years)	0-5	23 (23%)
		6-11	55 (55%)
		12-17	22 (22%)
3.	Type of epilepsy	Generalized epilepsy	78 (78%)
		Focal epilepsy	18 (18%)
		Unknown	4 (4%)
4.	Immunization status	Complete	74 (74%)
		Partial	19 (19%)
		Non-immunized	7 (7%)
5.	Number of drugs prescribed per patient	2	67 (67%)
		3	31 (31%)
		4	2 (2%)
		Mean ± SD	2.35 ± 0.52
		Median	2.00
		Range	2-4

**Figure 1:**
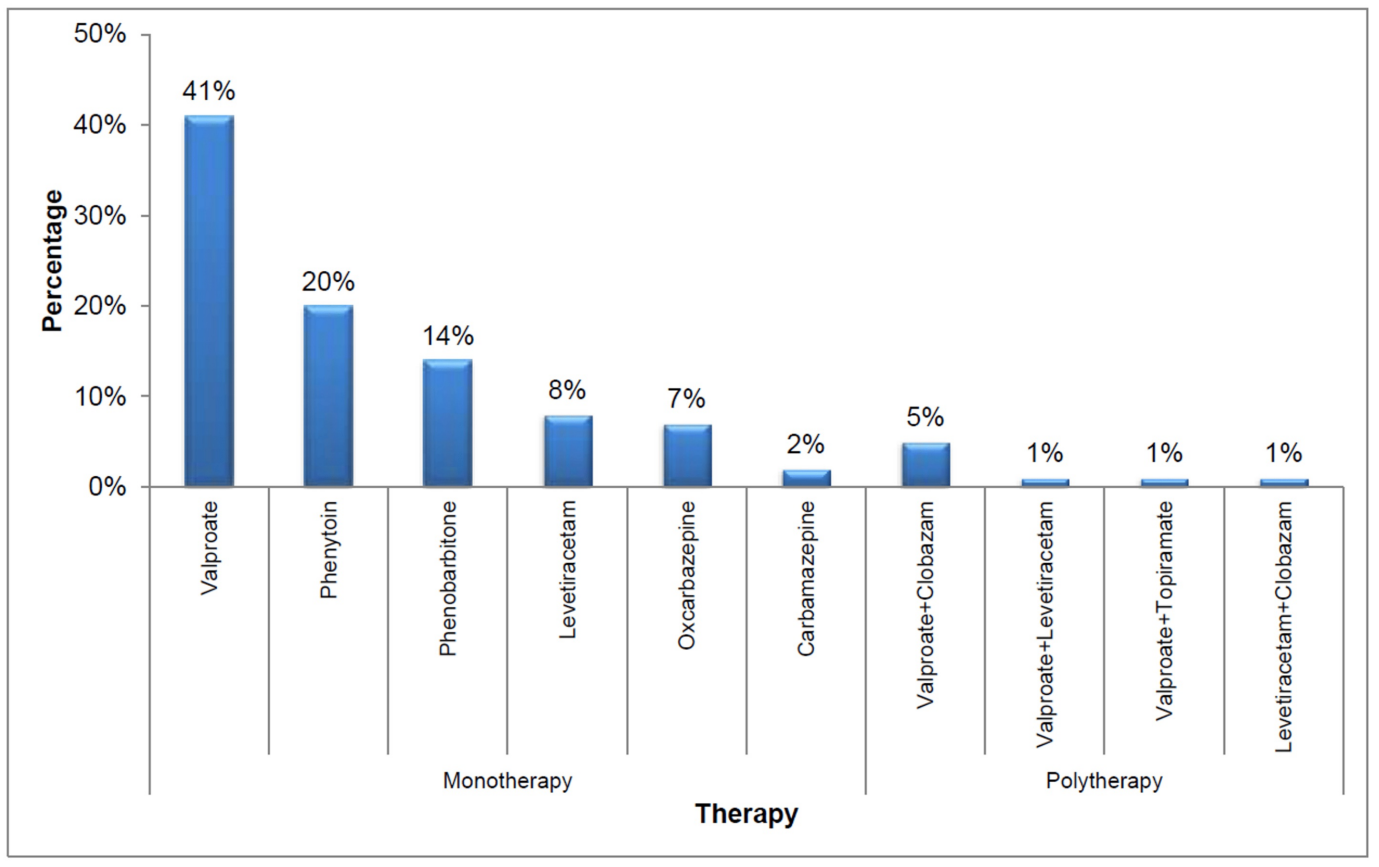
Distribution of drugs prescribed in monotherapy and polytherapy

Monotherapy (92%, n=92) was the predominant treatment approach, followed by polytherapy (8%, n=8). The distribution of individual drugs prescribed to patients undergoing monotherapy and polytherapy is illustrated in Figure 1.

The majority of patients (29%, n=29) had been on AED treatment for a duration of 7-12 months, followed by 13-18 months (21%, n=21), 19-24 months (22%, n=22), 3-6 months (17%, n=17), and more than 24 months (13%, n=13). A total of 67 ADRs were reported in 42 patients, with a higher predominance in males (71.43%, n=30). The maximum number of ADRs was reported in children belonging to the 6-11 years age group (68.65%, n=46) and those who have generalized epilepsy (73.13%, n=49). Patients on polytherapy (75%, n=6) reported a significantly higher number of ADRs (p=0.002) (Table 2).

**Table 2:** Distribution of ADRs based on type of therapy ADRs: adverse drug reactions

Type of therapy	Total number of patients	Number of patients who reported ADR (%)
Monotherapy	92	36 (39.13%)
Polytherapy	8	6 (75%)
X^2^	9.33	
p-value	0.002	

Among 42 patients, the majority of the population (52.38%, n=22) experienced a single ADR, followed by those who reported two (37.71%) and three ADRs (11.91%), respectively. All reported ADRs (100%, n=67) were classified as Type A ADRs according to the Rawlins and Thompson classification. Of the 67 ADRs, 53 (79.10%) were reported using the PESQ, while the remaining 14 (20.90%) were reported using methods other than the PESQ. The distribution of each ADR reported via PESQ is presented in Table 3.

**Table 3:** Distribution of ADRs reported using the PESQ ADR: adverse drug reaction, PESQ: Pediatric Epilepsy Side-effect Questionnaire​​​​​​​

Type of ADR reported	ADR reported	Number of patients (%)
A. Cognitive (43.39%) (total=23)	Slow thinking	0 (0%)
	Memory problems	4 (17.39%)
	Confusion	0 (0%)
	Poor school results	13 (56.52%)
	Decreased concentration	3 (13.04%)
	Attention difficulties	3 (13.04%)
B. Motor (0%) (total=0)	Unstable walking	0 (0%)
	Poor coordination/clumsiness	0 (0%)
	Falling (not seizures)	0(0%)
	Speech difficulties	0 (0%)
C. Behavioral (7.55%) (total=4)	Aggression	1 (25%)
	Hyperactivity	3 (75%)
	Personality change	0 (0%)
D. General neurological (33.96%) (total=18)	Drowsiness/sleepiness	9 (50%)
	Fatigue/tiredness	1 (5.56%)
	Dizziness/light-headedness	3 (16.67%)
	Headaches	5 (27.78%)
E. Weight (15.1%) (total=8)	Increase in appetite	4 (50%)
	Weight gain	4 (50%)

Apart from PESQ, 14 other ADRs were reported, comprising hair fall (50%, n=7), decreased appetite (21.43%, n=3), abdominal pain, and being irritable (14.29%, n=2 each). The distribution of ADRs, categorized by type of therapy and specific AEDs, is presented in Table 4.

**Table 4:** Distribution of ADRs reported in patients on monotherapy and polytherapy AEDs: antiepileptic drugs, ADRs: adverse drug reactions​​​​​​​

ADR reported	AEDs (monotherapy) ADR reported=56					AEDs combination (polytherapy) ADR reported=11	
	Valproate (32) (57.14%)	Phenytoin (10) (17.86%)	Phenobarbitone (7) (12.5%)	Oxcarbazepine (4) (7.14%)	Levetiracetam (3) (5.36%)	Valproate + clobazam (8) (72.73%)	Valproate + levetiracetam (3) (27.27%)
Memory problems	2 (6.25%)	__	1 (14.29%)	__	__	__	__
Poor school results	6 (18.75%)	3 (30%)	1 (14.29%)	__	__	2 (25%)	1 (33.33%)
Decreased concentration	__	1 (10%)	__	__	__	2 (25%)	__
Attention difficulties	2 (6.25%)	__	__	__	__	__	1 (33.33%)
Aggression	__	__	__	__	1 (33.33%)	__	__
Hyperactivity	__	__	3 (42.84%)	__	__	__	__
Drowsiness/sleepiness	4 (12.5%)	2 (20%)	1 (14.29%)	1 (25%)		1 (12.5%)	__
Fatigue/tiredness	__	1 (10%)	__	__	__	__	__
Dizziness/light-headedness	2 (6.25%)	1 (10%)	__	__	__	__	__
Headache	2 (6.25%)	__	__	3 (75%)	__	__	__
Increase in appetite	3 (9.37%)	__	__	__	__	1 (12.5%)	__
Weight gain	3 (9.37%)	__	__	__	__	1 (12.5%)	__
Hair fall	6 (18.75%)	__	__	__	__	1 (12.5%)	__
Decreased appetite	__	1 (10%)	1(14.29%)	__	1 (33.33%)	__	__
Pain abdomen	2 (6.25%)	1(10%)	__	__	__	__	__
Irritable	__	__	__	__	1 (33.33%)	__	1 (33.33%)

Additionally, 4.35% (n=4) of monotherapy patients and 37.5% (n=3) of polytherapy patients experienced seizure recurrence.

## Discussion

Epilepsy is a prevalent and significant neurological disorder characterized by seizures, contributing to considerable morbidity and mortality worldwide. It is estimated that over 10 million children globally are affected by epilepsy. The primary goal of AED therapy in children with epilepsy is to achieve seizure control, minimize drug-related side effects, and improve quality of life [8].

The present study reported a male predominance, aligning with the findings of Khoshdel et al., but in contrast to the observations of Sastry et al., who reported a higher prevalence among females [9,10]. Monotherapy was the most common treatment approach (92%), demonstrating its effectiveness in achieving good seizure control for the majority of patients. However, polytherapy was preferred for patients with poor seizure control. The predominance of ADRs in males was supported by a study conducted by Panda et al. [11]. Contrastingly, other studies indicated that females are more prone to developing ADRs [6,12]. An average of 1.59 ADRs per patient was observed, comparable to Kumar et al., who reported 1.96 ADRs per patient, but significantly lower than the 10.5 ADRs per patient reported by Egunsola et al. [13,14].

The predominance of ADRs in the 6-11 years age group and children with generalized epilepsy was consistent with findings by Bansal et al. and Jayalekshmi et al. [12,15]. The likelihood of developing ADRs increases with age, possibly due to changes in the pharmacokinetics and pharmacodynamics of AEDs [15]. Polytherapy was associated with a significantly higher rate of ADRs, which was supported by previous studies as well [6,16]. However, another study found no significant differences in adverse events between monotherapy and polytherapy and no correlation with AED load [17]. Factors influencing ADRs include individual reactions to treatment, the specific AEDs or combinations used, and the physician's expertise in managing therapy. Monotherapy remains the preferred approach due to its reduced side effects, absence of drug interactions, better compliance, lower cost, and improved seizure control in many cases.

Of the ADRs reported using PESQ, poor school performance was the most common ADR, similar to findings by Bansal et al. and Kaushik et al. [6,12]. However, other studies reported behavioral issues, somnolence, attention problems, decreased concentration, and irritability as the most frequent ADRs [14,18,19]. Cognitive ADRs (43.39%) were the most prevalent in this study, which aligns with the findings of Kaushik et al. However, Egunsola et al. and Junger et al. identified neurological and behavioral ADRs as the most common, respectively [14,20].

In monotherapy patients, valproate was associated with the highest number of ADRs, consistent with studies by Kaushik et al. and Jayalekshmi et al. [6,15]. Older/conventional AEDs were linked to more ADRs compared to newer drugs [21]. Valproate-related ADRs observed in this study align with findings by Yamak et al. [22]. Previous studies have observed that valproic acid may alter energy intake, physical activity, or both [23]. Phenytoin was noted for affecting mental speed and presented a high risk of cognitive effects, such as attention and memory issues, particularly at higher doses or in polytherapy [24]. ADRs associated with oxcarbazepine, including fatigue and headache, were consistent with findings by Donati et al. [25]. Levetiracetam was associated with aggression, decreased appetite, and irritability, consistent with previous studies [26,27]. No ADRs were reported in patients on carbamazepine, suggesting it was better tolerated.

In polytherapy, the combination of valproate and clobazam was associated with the highest number of ADRs. Previous studies also reported similar findings, including fatigue, drowsiness, slow thinking, ataxia, blurred vision, dizziness, emotional lability, irritability, depression, weight gain, and memory issues with clobazam [14,28]. The recurrence of seizures, especially in patients on polytherapy, was a concern. Komatsubara et al. identified polytherapy, comorbid conditions, therapy discontinuation, and other factors as contributing to seizure recurrence [29]. Additional factors may include non-compliance, abrupt AED changes, inappropriate dosing, or triggers such as sleep deprivation, febrile illness, or concurrent medications. As all the reported ADRs were classified as Type A according to the Rawlins and Thompson classification, none of the patients required discontinuation of antiepileptic therapy. Detailed analysis of prescription patterns based on the latest National List of Essential Medicines 2022, use of a validated questionnaire to study ADRs​​​​​​, reporting of ADR other than by PESQ, and information about recurrence of seizures constituted the strengths of the present study. Thus, this study will be useful in encouraging rational prescribing by physicians in the pediatric population. The main limitations of our study include its cross-sectional design and small sample size, which limit generalizability. ADRs were verbally reported without any formal assessment of cognitive or behavioral effects; thus, recall bias may have influenced responses. Although a single interviewer was used to ensure uniformity, the possibility of observer bias cannot be entirely ruled out.

## Conclusions

ADRs were more common in patients on conventional drugs when used as monotherapy as well as in combination. Use of newer drugs by the prescribing physicians, as well as self-reporting of ADRs by the patients, should be encouraged for the comprehensive management of pediatric epilepsy.
